# The Foreign Journals: Midwifery

**Published:** 1840-07

**Authors:** 


### MIDWIFERY.

### *Case of a Dwarf in whom Premature Labour was artificially induced with success.* By M. Paul Dubois.

C. L. was born in Italy, from a mother of ordinary stature and a father only three feet and a half high, though straight and well-proportioned, who was himself the son of a dwarf, and had three dwarf sisters. He had six children, of whom three were born and remained dwarfs, and three were born well-developed and acquired the ordinary stature.

Caroline (the subject of the present observations) presented in her early infancy nothing remarkable except her extreme diminutiveness. She was brought up with facility; she menstruated without difficulty when eleven years old, and the catamenial function has since been regularly performed, the quantity of fluid discharged being nearly the same as in a woman of ordinary size. When twelve years old she was brought to France with one of her dwarf brothers, and both were for some time publicly exhibited. At the conclusion of this engagement, when about twenty years old, she formed a connexion with a man of ordinary stature; the sexual intercourse was at first extremely painful, but she soon became pregnant. She went to the full period of gestation without anything remarkable occurring, and on the 3d of April, 1838, the first labour-pains occurred.

At first the labour presented nothing peculiar; the pains were regular, the dilatation of the os uteri complete, the membranes burst of their own accord, and well-sustained expelling efforts continued without result till the next day. On the 5th she was attacked with convulsions, which were repeated several times, and produced first an occasional and then a permanent state of stupor. A physician was called in, but considering that the expulsion of the child could not be effected without artificial aid, he called in M. P. Dubois.

The little patient was then lying in a cradle in that sleepy state which succeeds to attacks of eclampsia, and occupies the intervals between them; there was complete abolition of sensation and intelligence, and relaxation of the limbs. The child's head was found partially engorged in the upper aperture of the pelvis, projecting into its hollow; the os uteri was completely dilated; and having ascertained that the child was dead, M. Dubois determined on applying the forceps. Notwithstanding the apparent disproportion between their size and that of the patient, they were easily though unsuccessfully applied; but without removing them, M. Dubois introduced between their blades a pointed bistoury, with which he perforated the child's head. The greater part of the brain was at once evacuated, and the head passed on to the vulva; but the small size of that aperture rendered its rupture inevitable, and it tore obliquely towards the right buttock round the anus, but leaving the sphincter uninjured.

The child without its brain weighed five pounds and a half, and was seventeen Paris inches and a half long. The mother remained in the same sleepy state till the next day, and then on recovery had no recollection of anything that had passed; the delivery was followed by some inflammatory symptoms, but they yielded to appropriate treatment.

Before leaving her, M. Dubois ascertained that the antero-posterior diameter of the upper part of the pelvis was only nearly three inches; he therefore told her her danger, and advised her if she again became pregnant to come to him very early. In September, 1839, she wrote to him that she was again pregnant, and with some difficulty the date of her conception was fixed at the early part of June; she was carefully watched during the following months, and on the 13th of February, 1840, the uterus having in the few preceding days increased considerably in size, M. Dubois determined to excite the contractions of the uterus.

At half-past nine in the morning, a common speculum being passed into the vagina, a piece of prepared sponge, about an inch and a half long, and cone-shaped, with a base from six to eight lines in diameter, was introduced by means of a long pair of forceps into the os uteri. It was fixed in its place by a second larger piece, and threads were adapted to both so that they might be drawn out as soon as it was necessary. The patient was scarcely placed in her bed again before she felt some slight pains; forty grains of ergot of rye were almost directly after administered in doses of five grains every ten minutes; and during its administration the pains became gradually stronger and longer, and from eleven to twelve o'clock followed each other very rapidly and were well-sustained.

At half-past twelve the sponges were withdrawn, and that in the neck of the uterus was found of four times its previous size. The orifice would easily have admitted the tips of two fingers, but as it was rather rigid, some extract of belladonna was applied to it. The pains continued increasing; and at half-past one the membranes could be reached though rendered too tense by the great quantity of liquor amnii to permit the mode of presentation to be detected. The pulse was regular, at ninety-two. From half-past two to half-past three the pains diminished; they then again returned but at long intervals, during which the patient fell into a state of profound sleep. At half-past four she was bled to four ounces and the pains soon became stronger. At half-past six she had nausea and vomited some mucus containing a little ergot of rye. From that time the pains ceased, and the patient slept calmly till eight in the evening, when a few slight uterine contractions again took place.

The dilatation of the os uteri was scarcely altered, and the cervix retained nearly the same length up to eight o'clock. The membranes were then ruptured and a great quantity of fluid evacuated; the fœtus approached the orifice of the uterus, and the buttocks were found presenting. From this time the pains were regular; at eleven the cervix was almost completely obliterated, and at midnight its dilatation was nearly perfect. At one o'clock the pains were less vigorous and frequent, and forty grains (five every ten minutes) of ergot of rye were administered, but were nearly all rejected by vomiting. At three the same quantity was administered in an enema; the uterine contractions then returned with full force, and by seven in the morning of the 14th the buttocks presented at the vulva. M. Dubois now hooking his fore-finger round one of the haunches, extracted the trunk without difficulty, and then disengaged the arms. The head was for some time arrested at the upper strait of the pelvis; tolerably powerful traction, during which the infant inspired several times, was necessary; and at last the head passed through and the child was in another instant born.

It was a little girl, which was at first weak but very quickly recovered. The expulsion of the placenta was followed by rather considerable hemorrhage, but it was checked by friction. The mother's recovery was rapid and natural.

The infant at birth weighed three pounds and three quarters; its total length was fifteen inches; from the vertex to the umbilicus it was eight inches and one sixth; the occipito-frontal diameter of the head was three inches and a half; the occipito-mental was four inches and a quarter; the bi-parietal three inches; the sub-occipito-bregmatic three inches and one eighth. The mother was three feet three inches high, and on the whole well proportioned.

*Bulletin de l'Académie Royale de Médecine. Mars* 31, 1840.

---

### *On the Funic Bellows-sound.* By Dr. Dietrich.

In addition to the placental murmur and those accompanying the pulsations of the fœtal heart, there is a third sound occasionally to be heard in pregnant women, and one which is well ascertained to have its seat in the umbilical cord. It is a single murmur of the bellows' species, and synchronous with the first sound of the fœtal heart. Dr. Dietrich is of opinion, herein agreeing with others who have examined this subject, that the sound depends on diminution of the caliber of the umbilical arteries either through pressure or stretching of the funis or both combined. He gives cases illustrating its production under these different conditions. In the first, wherein the bellows-murmur is presumed to have been the effect of pressure, the umbilical cord was of the unusual length of twenty-four inches and a half, twisted once round the neck and tightly knotted in its middle third; the knotted part having acquired from its tightness a white, shining, tendinous appearance. In two cases exemplifying the occurrence of the sound in consequence of a tense state of the cord, this was only ten and fourteen inches long, and this unusual shortness led to greater tension in the former instance from the presentation being a breech one; in neither case was the cord twisted round the neck. In the example of production of the sound from pressure and stretching combined, the navel-string was twenty-three inches long, of normal structure, and wound twice round the neck; the accoucheur was obliged to cut it during delivery, finding it impossible to slip it over the head. Here the peculiar sound was invariably heard on every examination; an unusual circumstance attributed to the tightness with which the cord was twisted, whereby change of position or other occurrences, sometimes causing a temporary cessation of the sound, were rendered of none effect.

*Medicinische Zeitung.* No. xxxvii. 1839.

---

### *On Compression of the Abdominal Aorta in cases of Violent Flooding after Delivery.* By Dr. Ehrenreich.

A woman after having been in labour for thirty-four hours, and the liquor amnii discharged for thirty, was then, on account of narrowness of the pelvis and the absence of pains, delivered with the forceps of a healthy living girl, her first child. The separation of the placenta soon followed; not a particle of it was left behind, and the uterus contracted regularly. A very moderate quantity of blood had been lost during and immediately after delivery. Two hours after I was hastily summoned, and found the woman in a fainting state, the countenance collapsed, and of death-like paleness; the pulse scarcely perceptible; the hands and feet cold; the voice almost gone. On examination I ascertained that the uterus was relaxed, and that the woman, so to speak, floated in blood. As the ordinary means for stopping hemorrhage were insufficient in this case, I determined to try compression of the aorta, and accordingly introduced the entire right hand into the uterus, and with the four fingers gathered into a conical shape made forcible pressure on the vessel immediately above its bifurcation. The bleeding ceased completely. At the same time I stimulated the walls of the uterus by moving my thumb, and rubbed the corresponding part of the abdomen with the left hand. During the continuance of the compression analeptics with tincture of opium and cinnamon were administered, and as soon as the patient was somewhat restored, secale cornutum in fifteen grain doses every half hour. Cold poultices were also applied to the abdomen and subsequently sprinkled with æther. All this, however, produced no contraction of the uterus, and the moment I ceased to press on the aorta, the bleeding burst out afresh. After having kept up the compression for nearly half an hour, my hand and arm were completely disabled, and I was unable to press with sufficient force. A change of hands would have caused too great a loss of blood; I therefore determined to lay a heavy sand-bag, as recommended by Kluge and Betschler, on the part of the abdomen corresponding to the uterus—a plan of which I had previously ascertained the utility. This was done, while I gradually withdrew my hand; the moment the bag was well placed all the bleeding ceased, which had returned after the removal of my hand. In two hours after, the skin had recovered its warmth, and every bad symptom disappeared: the bag was then taken away, having been gradually lightened by removal of its contents. Six weeks after the patient was perfectly well with the exception of weakness; she suckled her infant herself.

[External compression with the hand on the abdomen, recommended by others, and the only way of acting on the aorta when an irregularly contracted state of the uterus may prevent introduction of the hand, was first tried in this case; but from the thickness of the abdominal parietes it had no control over the bleeding.] *Medicinische Zeitung.* No. xxxvii. 1839.

---

*On Trismus in New-born Infants.* By Dr. C. E. LEVY, Resident Physician at the Foundling Hospital in Copenhagen.

DR. LEVY refers this disease to inflammation of the umbilical arteries, and relates six cases in confirmation of this opinion which had previously been expressed by Professor Busch, of Berlin. The cases exhibit the remarkable rapidity with which this inflammatory process passes into the suppurative stage and occasions destructive ulceration of the coats of the arteries. The following are the principal facts of Dr. Levy's cases:

| Case | Cord separated | Illness began | Death | Post-mortem Examination. |
|---|---|---|---|---|
| 1 | 4th day | 6th day | 7th day | Suppuration of the arteries with loosening and discoloration of their lining membrane. |
| 2 | 5 | 6 | 7 | Extensive ulcerative destruction of the coats of the arteries. |
| 3 | 4 | 3 | 8 | Destructive suppuration with perforation of the right umbilical artery. |
| 4 | 5 | 5 | 7 | Suppuration with discoloration, and partial destruction of the lining membrane of the arteries. |
| 5 | 5 | 5 | 6 | Suppuration, with discoloration and loosening of the lining membrane of the arteries. |
| 6 | 3 | 3 | 5 | Destructive suppuration, with softening and perforation of the walls of the arteries. |

The diseased condition of the umbilical arteries did not in any case extend up to the point where they unite with the hypogastric arteries.

*Neue Zeitschrift für Geburtskunde.* Band vii.

